# Barriers to and facilitators of gestational diabetes mellitus service utilization in Kenya: a qualitative analysis of women’s and healthcare workers’ perspectives

**DOI:** 10.1186/s12913-026-15981-2

**Published:** 2026-10-31

**Authors:** Angela Obongo, Hannah Eger, Marie Tallarek, Jacob Spallek

**Affiliations:** 1 Department of Public Health, Faculty 4 Faculty of Human Sciences, Brandenburg University of Technology Cottbus-Senftenberg, Universitätsplatz 1, 01968 Senftenberg, Germany; 2 Médecins du Monde Germany, Berlin, Germany

**Keywords:** Gestational diabetes mellitus, Maternal health, Access to care, Sociocultural barriers, Health equity

## Abstract

**Background:**

Gestational diabetes mellitus (GDM) is a growing global public health concern, particularly in low- and middle-income countries such as Kenya. Despite rising prevalence and associated maternal and neonatal risks, timely diagnosis and comprehensive GDM care remain limited by systemic, socioeconomic, and cultural barriers. This study identified barriers and facilitators influencing GDM care provision and utilization in Kisumu County, Kenya, from the perspectives of healthcare workers (HCWs) and women affected by GDM.

**Methods:**

A qualitative content analysis combined semistructured interviews and structured surveys. Participants were purposively sampled from two healthcare facilities in Kisumu County: Jaramogi Oginga Odinga Teaching and Referral Hospital and Kisumu Specialists Hospital. Interviews involved eight HCWs and two women diagnosed with GDM, and surveys were administered to eleven women with a history of GDM. Data collection instruments, developed from the health needs assessment and knowledge, attitude, and behaviour frameworks, were validated by an expert panel from Kenya and Germany. Thematic analysis identified key barriers to and facilitators of GDM care.

**Results:**

Stakeholders highlighted systemic gaps and resource constraints affecting GDM care, including high costs, inadequate insurance, supply shortages, and poor infrastructure, which especially affected rural and low-income women. Barriers including financial burden, distance, inconsistent protocols, communication gaps, and negative patient-provider relationships hindered access and adherence. Facilitators included family and social support, health worker education and follow-up, and self-management through dietary counseling, monitoring, and lifestyle changes. All groups advocated universal screening, expanded insurance, community outreach, and better education to strengthen GDM care and improve outcomes.

**Conclusions:**

GDM care provision and utilization in Kenya are shaped by multilevel barriers spanning individual, systemic, and cultural domains. Addressing them requires targeted interventions, including enhanced HCW training, greater community awareness, and strengthened health system infrastructure. Integrating GDM services into existing maternal health programmes is essential to improving outcomes for women and their infants in resource-constrained settings.

## Background

Gestational diabetes mellitus (GDM), defined as any degree of hyperglycaemia with onset or first recognition during pregnancy (typically after the 24th week), is a significant and growing global public health concern. Hyperglycaemia affects approximately 28% of pregnancies globally, with GDM accounting for nearly 90% of these cases [1–4]. According to the International Diabetes Federation (IDF) 2021 report in Diabetes Research and Clinical Practice, the global age-standardized prevalence of GDM is approximately 14.0%, with marked regional disparities. Prevalence was 7.1% in North America and the Caribbean and 7.8% in Europe, but higher in South and Central America (10.4%), Africa (14.2%), and the Western Pacific (14.7%). Rates were elevated in Southeast Asia (20.8%) and highest in the Middle East and North Africa (27.6%). By economic classification, prevalence was 12.7% in low-income, 9.2% in middle-income, and 14.2% in high-income countries. These pronounced geographic and socioeconomic disparities underscore the need for tailored public health interventions in screening, prevention, and management across diverse global contexts [5]. Notably, approximately 90% of GDM cases occur in low- and middle-income countries (LMICs), where health systems face significant strain from the concurrent burden of communicable and noncommunicable diseases (NCDs) [6–8].

In Kenya, NCDs like diabetes are a major public health concern, causing over 50% of hospital admissions and 55% of deaths. Adult diabetes prevalence is 4.56%, with urban rates (10.7%) far exceeding rural rates (2.7%). Over 60% of cases are undiagnosed, often discovered incidentally during unrelated visits, suggesting current estimates understate the crisis. A further 14% of Kenyans have impaired glucose tolerance, indicating growing GDM risk [9]. GDM has a multifactorial etiology, with risk factors including advanced maternal age, family history of type 2 diabetes mellitus (T2DM), previous macrosomic delivery (≥ 4 kg), and prepregnancy overweight or obesity. It is associated with adverse maternal and neonatal outcomes, including preeclampsia, preterm birth, polyhydramnios, fetal macrosomia, cesarean delivery, and perinatal mortality. Although GDM typically resolves after delivery, it substantially increases the long-term risk of T2DM in affected women and causes metabolic disturbances in offspring, including obesity and impaired glucose tolerance [2, 6, 8, 10–14]. In sub-Saharan African countries (SSACs), addressing GDM requires context-specific strategies informed by local epidemiology and risk factors. Key interventions include early diagnosis, lifestyle adjustment, medical treatment, antenatal monitoring, and postpartum follow-up to prevent progression to T2DM. GDM care offers a critical platform for health education, glycemic management, and sustainable lifestyle changes, such as better nutrition and physical activity, with long-term maternal and child health benefits. Postpartum counseling is especially vital for reducing future T2DM risk [2, 15, 16]. Effective management depends on timely diagnosis and comprehensive care, including evidence-based lifestyle interventions, regular blood glucose monitoring, and pharmacological therapy when indicated [2, 17]. However, access to such services remains limited in many LMICs, including Kenya, due to systemic, socioeconomic, and cultural barriers [7, 14, 18–22].

In Kenya, access to maternal healthcare services (MHCS), including GDM care, reflects a complex interplay of systemic, socioeconomic, and cultural factors. Despite advances in maternal health, significant challenges persist, including regional inequities, shortages of trained personnel, and constrained diagnostic capacity [20]. These gaps are compounded by financial barriers, geographic inaccessibility, and deeply rooted social norms, contributing to disparities in the access to and quality of maternal and GDM-related care [21–27]. Knowledge and awareness of GDM are also often low among pregnant women in SSACs, hindering timely detection, appropriate management, and prevention of complications [22, 27–29].

### Structure of Kenya’s healthcare system

Kenya’s healthcare governance and service delivery are shaped by the devolved structure introduced under the 2010 Constitution, which divides responsibilities between national and county authorities. The National Government leads strategic direction, including health policy, regulatory oversight, capacity building, resource mobilization, research coordination, management of national referral hospitals, and technical assistance to counties. County governments directly implement services, managing local health facilities (clinics, dispensaries, hospitals) and pharmacies, operating ambulances, advancing primary healthcare, and regulating food safety and hygiene. Kenya’s health services are organized into a six-level hierarchy of escalating care complexity. Level 1 comprises community health services emphasizing preventive and promotive care through community health units. Level 2 dispensaries provide basic outpatient services, diagnostics, and treatment for minor ailments. Level 3 health centres offer comprehensive outpatient care, basic maternity services, and limited inpatient care. Level 4 county hospitals serve as primary referral facilities with inpatient and specialized outpatient services. Level 5 county referral hospitals deliver advanced care, including specialist consultations and complex procedures. Level 6 national referral hospitals provide highly specialized services and serve as centres for clinical training and research. This tiered framework supports an integrated approach aligned with Kenya’s devolved governance model [20, 21]. Despite these structures, disparities in access and quality persist across regions. Maternal and child health initiatives are hindered by shortages of trained personnel, inadequate supplies, and geographic inequities [20, 21]. Sociocultural norms, high costs, and long travel distances further restrict access to essential GDM services in rural and marginalized communities [21, 22, 27]. Kenya’s National Health Insurance Fund (NHIF), established in 1966, was the country’s primary public health insurer for over five decades. In 2023, Parliament enacted the Social Health Insurance Act, 2023, repealing the NHIF Act and establishing the Social Health Authority (SHA) as its successor, which commenced operations on October 1, 2024 [30].

This study is the second phase of a larger project on GDM care delivery in Kenya. The first phase used policy expert consultations and analysis of national health documents from Kenya’s Ministry of Health (MoH) to assess how GDM is framed within Kenya’s NCD policies [31]. Data from both phases (Table 1) are cross-referenced and discussed. Focusing on systemic challenges, this phase aimed to identify barriers and facilitators affecting GDM care provision and uptake, hypothesizing that structural factors significantly shape healthcare worker (HCW) and patient experiences. By integrating insights from HCWs and women with GDM, the findings aim to guide policies and interventions that strengthen GDM services in Kenya and comparable low-resource settings.

**Table 1:** Cross-cutting patterns and thematic correlations

Cross-cutting pattern	Key findings	Implication for policy and practice
Financial constraints and insurance	Cost barriers are a critical hindrance to GDM care. Policy experts and providers note affordability issues, and patients struggle with the high costs of tests, medication, and monitoring supplies.	Expansion of NHIF/UHC coverage for GDM-related services to eliminate financial barriers. Consider free or subsidized screening and treatment for pregnant women.
Resource and infrastructure gaps	Limited availability of essential equipment (glucometers, OGTT labs, insulin), particularly in rural and lower-level facilities. Urban hospitals are better equipped, but shortages still occur.	Increased funding and procurement of necessary medical supplies for diabetes management, especially at county hospitals and lower-tier facilities. Strengthen supply chain management.
Policy and guidelines vs. practice	Lack of a dedicated diabetes policy; GDM guidelines exist but are not detailed or well-disseminated. Providers report inconsistent protocols, and patients notice variability in advice given at different facilities.	Development of a standalone diabetes policy or more comprehensive GDM guidelines with clear protocols for screening, diagnosis, and management. Improve dissemination and enforcement.
Knowledge and awareness gaps	Low awareness of GDM among healthcare workers, patients, and policymakers. Knowledge gaps contribute to poor adherence to management plans and may not reflect the true prevalence in the community. Training and public awareness campaigns are needed.	Strengthen education initiatives for both providers and patients. Include GDM in routine antenatal health talks and launch community awareness campaigns.
Patient-provider interaction and attitudes	Providers perceive patients as noncompliant, while patients feel providers are sometimes dismissive or unclear in their communication. Poor communication leads to misunderstandings and nonadherence.	Improve provider communication skills training to foster empathy and patient-centered care. Encourage patient feedback mechanisms to enhance trust and adherence.
Geographical disparities	Rural patients face difficulty reaching specialized GDM services due to distance and transportation costs. Referral systems are weak, making follow-ups challenging. Decentralized screening and treatment are needed.	Implement mobile clinics and outreach programs to improve rural access. Strengthen referral networks to ensure patients diagnosed with GDM receive consistent follow-up care.

Table 1 summarizes overarching themes of the barriers and enablers to accessing GDM services in Kenya, drawn on insights from policy experts at the Kenyan MoH, healthcare providers, and patient perspectives

## Methods

A qualitative content analysis (QCA) approach captured the lived experiences of key stakeholders and elucidated systemic challenges in GDM care pathways. The health needs assessment (HNA) and knowledge, attitudes, and behaviours (KAB) frameworks guided development of the data collection instruments (Tables 2, 3 and 4) [31].

**Table 2:** Integration of the HNA and KAB frameworks in the survey for women with a history of GDM

Integrated framework component	Survey questions
Health status & risks (epidemiological + knowledge)	“Q15: Have you ever been tested with gestational diabetes before or during pregnancy? Q18: Do you have a family history of diabetes?”
Access to care & treatment (comparative + knowledge + attitude)	“Q15a-c: If you answered yes to been tested with gestational diabetes before? in which facility were you treated? how were you treated; which medicines did you take? Q16: Have you used alternative medicine?”
Social & cultural impact (corporate + attitude)	Q3: What is the highest level of formal education you have attained? Q6: Please specify your cultural or ethnic identity (e.g., tribe, community). Q7: If applicable, what is your partner’s cultural or ethnic identity (e.g., tribe, community)? Q8: How many individuals currently reside in your household, including yourself? Q9: Who is the primary financial provider in your household?
Patient experience & self-management (needs + behavior)	Q26-Q35: Cognitive domain – assessing factual understanding of appropriate management practices and health literacy – reflecting the respondent’s knowledge of lifestyle and medical treatment requirements for GDM
Healthcare system & Policy challenges (comparative + corporate + knowledge + behavior)	Q10-Q14: Pregnancy and GDM impact on daily life Q22-Q24: Symptoms and complications of GDM

The expected outcomes were knowledge gaps, attitudes toward treatment, and actual health behaviors. Individual behaviours were mostly assessed [31]

**Table 3:** Integration of the HNA and KAB frameworks into the interview guide for HCWs

Integrated framework component	Interview questions
Health status & risks (epidemiological + knowledge)	Q2: what are the procedures or criteria used to diagnose GDM? Q3: What are the typical characteristics of patients who seek GDM care at the facility?
Access to care & treatment (comparative + knowledge + attitude)	Q4: What treatment options are available? Q6: To what extent do patients understand self-management of GDM, and how do they interpret or approach it?
Social & cultural impact (corporate + attitude)	Q7: How do patients react to a GDM diagnosis? Q11: Do cultural differences affect care delivery?
Patient experience & self-management (felt needs + behavior)	Q9: What challenges do you face in treating GDM patients? Q13: How do you handle patients struggling with adherence?
Healthcare system & policy challenges (comparative + corporate + knowledge + behavior)	Q10-Q12: What are the biggest obstacles in GDM care delivery?

The expected outcomes were structural barriers in healthcare services and aimed to capture knowledge gaps among providers and attitudes toward patient behavior [31]

**Table 4:** Integration of the HNA and KAB frameworks into the interview guide for women with GDM

Integrated framework component	Interview questions
Health status & risks (epidemiological + knowledge)	“Q1: When was your GDM first diagnosed? Q9: How do you think GDM will affect your unborn child?”
Access to care & treatment (comparative + knowledge + attitude)	“Q10-Q14: How was your contact with the healthcare facility where you received your diagnosis or go for treatment? Describe the information you received during the consultations?”
Social & cultural impact (corporate + attitude)	“Q6-Q7: How did your partner react to your diagnosis (if applicable), was he understanding for example? Which positive support did you receive and which negative support did you notice?
Patient experience & self-management (needs + behavior)	“Q15-Q20: What do you do daily to manage GDM? What are the challenges you face regarding your treatment?”
Healthcare system & policy challenges (comparative + corporate + knowledge + behavior)	“Q22-Q28: What have been the challenges you have faced during the management of your condition? Describe what is making you adhere or not to adhere to your post diagnosis diet and physical activity program.”

The expected outcomes were attitudes toward self-management and treatment adherence and aimed to capture both patient knowledge and systemic barriers to care [31]

### HNA theory

HNA is a systematic process for optimizing the allocation of healthcare resources to improve population health outcomes. Because providers traditionally assess individual health needs during clinical consultations, this method may overlook broader community health issues. People with chronic illnesses or socioeconomic disadvantage often avoid medical care, leaving community health concerns unaddressed. Historically, equitable resource allocation initiatives prompted by structural adjustments have underscored the importance of addressing these disparities. Today, amid rising healthcare costs, resource limitations, and uneven geographical service distribution, HNA remains pivotal for distinguishing between health needs, public demands, and resource availability, ensuring practical and efficient interventions [32]. Individual beliefs and values significantly shape health-seeking behaviors, which are influenced by personal understanding, cultural learning, and interpersonal networks [33–35]. Sociodemographic and economic contexts further influence health decisions, emphasizing the importance of considering broader environmental determinants when analysing healthcare utilization [35]. In resource-constrained settings, health policies often reflect Western paradigms, sometimes at the expense of indigenous practices and localized health needs. These models typically emphasize treatment-oriented, hospital-centric care, potentially neglecting traditional or locally informed approaches [32]. Addressing these disparities requires structured needs assessments that systematically identify, analyse, and prioritize population-specific healthcare requirements, enabling tailored and effective interventions [32, 36].

### KAB

Knowledge shapes health behaviors by informing attitudes and actions. KAB describes how greater health awareness gradually shifts attitudes, guiding health-related decisions and adherence to recommended practices [37]. Attitudes, as personal perceptions of health behaviors, determine receptiveness to new practices, with positive attitudes fostering healthier choices [37, 38]. Behavioral change thus results from evolving attitudes, positioning KAB as a cognitive strategy for designing educational interventions to improve long-term outcomes [38, 39].

By integrating the HNA and KAB frameworks, this study evaluated health needs, perceptions, and behaviors in individuals with GDM, yielding insights into knowledge-attitude-behavior dynamics to inform interventions and policies. Survey and interview tools were validated by an expert panel (three from Germany, three from Kenya) specializing in obstetrics, public health, epidemiology, and qualitative research. Their feedback ensured content validity, cultural appropriateness, and clarity, refining question structure, wording, and relevance to enhance cross-cultural applicability and usability for respondents and providers.

### Study design and participants

A QCA explored the experiences, perceptions, and challenges related to GDM care among HCWs and women diagnosed with GDM in Kisumu County, Kenya. Data collection used semi-structured interviews and a structured survey across two facilities, one public and one private. Interviews involved 10 participants: 8 HCWs and 2 women diagnosed with GDM. HCWs were drawn equally from Jaramogi Oginga Odinga Teaching and Referral Hospital (JOOTRH), a public Level 6 referral and teaching hospital (J1–4), and Kisumu Specialists Hospital (KSH), a privately operated Level 4 county referral facility (K1–4). The two patients with GDM were recruited from JOOTRH (JP) and KSH (KP), respectively. Interviews aimed to elicit in-depth insights into barriers to and facilitators of GDM service delivery, patient experiences, and strategies for improving care. The survey was administered to 11 women with a history of GDM, all receiving care at JOOTRH. Both facilities were selected purposively for their roles in maternal and diabetes care at the county level and for their financial feasibility, administrative accessibility, and geographic relevance.

In the project’s initial phase, consultations with national-level experts revealed substantial gaps in GDM data and epidemiological information. Experts noted that GDM data collection has been constrained by technical limitations in health information systems and inconsistent reporting. Anticipated bureaucratic hurdles were also expected to affect the timeline and budget. These challenges were compounded by lingering Coronavirus disease (COVID-19) public health restrictions, as data collection was scheduled to begin in February 2022. A purposive sampling strategy was therefore adopted to include relevant stakeholders and contexts under these constraints.

Interviews were conducted in private facility spaces to ensure confidentiality and minimize disruptions. All participants provided prior written informed consent, and interviews were audio-recorded with their permission.

Data collection instruments: To understand how women experience GDM, seek treatment, and are supported by HCWs and policies, semi-structured interview guides and survey questions were formulated, drawing insights from women with GDM and healthcare professionals. The HNA and KAB frameworks guided the design of these tools, providing a systematic approach to analysing individuals’ perceptions of health, healthcare-seeking behaviours, and engagement in health-related activities. The interview guides were developed to assess health system deficiencies by identifying barriers to care for individuals with GDM, to examine determinants of behavioural change by exploring knowledge gaps, attitudes, and treatment adherence, and to inform policy and health system improvements. The study used two instruments: a structured survey for women with a history of GDM and semi-structured interviews with women diagnosed with GDM and HCWs.

### Sampling strategy and language considerations

Given the limited GDM data, purposive sampling was the most appropriate recruitment approach. Eligible HCWs were invited through the maternal and child health services (MCHS) department. Patients with GDM were identified by MCHS staff and approached only after expressing initial interest through the MCHS team, ensuring voluntary consent. Interviews were conducted primarily in English, with Swahili offered to accommodate participant preferences and aid comprehension. Data collection took place between February and June 2022.

### Inclusion and exclusion criteria

#### Inclusion criteria

##### HCWs

- Licenced medical professionals (doctors, nurses, or midwives) currently engaged in MHCS or diabetes/endocrinology services.
- Employed at either JOOTRH or KSH during the study period.
- Provided informed consent and were willing to participate in interviews discussing their clinical roles and experiences with GDM care.

##### GDM patients

- Women aged 18 years and above with a clinically confirmed diagnosis of GDM.
- Either undergoing current treatment or with a prior history of GDM management.
- Willing to provide informed consent and participate in discussions regarding diagnosis, treatment, symptom management, health system interactions, and coping strategies.

#### Exclusion criteria

##### HCWs

- Administrative staff without direct clinical responsibilities.
- Individuals who declined consent or were unwilling to participate in interviews.

##### GDM patients

- Women without a formal GDM diagnosis.
- Those diagnosed with preexisting type 1 diabetes mellitus or T2DM, due to differing disease trajectories and treatment pathways.
- Individuals under 18 years of age.
- Those with severe psychiatric conditions, cognitive impairments, or other medical conditions precluding effective participation.
- Individuals who refused consent, withdrew from the study, or expressed discomfort with sharing personal health information.

### Data coding and analysis

Both the interview and survey data were analysed via QCA following the methodological framework outlined by Mayring [40]. The analysis was supported by MAXQDA Analytics Pro, version 24.5.1. All interview data were transcribed verbatim and anonymized by AO to protect participant confidentiality. The process began with data familiarization, reviewing transcripts multiple times to understand content and context. Meaning units and text segments relevant to the research objectives were then identified and systematically coded, using a deductive approach to category assignment drawn from pre-established theoretical constructs relevant to GDM care.

To guide coding, a framework was developed and documented in a coding manual containing category definitions, coding rules, and illustrative anchor examples to promote consistency, clarity, and reliability. Initial categories were informed by literature on GDM service delivery and health systems research. In the first coding phase, these categories were applied across all transcripts, with meaning units assigned accordingly. As analysis progressed, the framework was refined to resolve ambiguities or discrepancies in category application. Where meaning units did not fit existing categories, new subcategories captured emergent themes. Overlapping categories were consolidated and definitions revised for internal consistency and theoretical coherence. To enhance reliability and credibility, intercoder agreement checks were conducted at two stages: two researchers (AO and HE) independently coded a subset of transcripts, and discrepancies were resolved through discussion, refining coding definitions and rules. Intercoder agreement was reached through structured consensus discussion. Once stable, the framework was applied to the entire dataset. Analytical rigor was maintained through peer debriefing, reflexive practices, and member checking, consistent with qualitative criteria for credibility, transferability, and confirmability. Member checking involved three HCWs who reviewed preliminary findings and confirmed the accuracy of thematic interpretations, contributing to validation and refinement. Peer debriefing sessions with five independent healthcare and public health experts from Germany and Kenya reviewed the study design, coding strategy, and preliminary analysis, informing adjustments to thematic categories and ensuring reflexivity and analytic depth. The structured survey comprised categorical (correct, incorrect, uncertain) and multiple-choice items on GDM risk factors, symptoms, complications, treatment modalities, and sources of social support. Responses were grouped into thematic domains, including risk awareness, symptom recognition, treatment adherence, and misconceptions. Correct responses were aligned with established clinical guidelines, while incorrect and uncertain answers were analysed to identify knowledge gaps. Responses were further examined for sociodemographic influences on health literacy and misconceptions about GDM.

### Ethical approval

This study was conducted in accordance with the Declaration of Helsinki. Its design, data collection, and dissemination adhered to the principles of respect for persons, beneficence, and justice for research involving human participants. Ethical clearance was obtained from the institutional ethics review boards of Maseno University (Kenya), JOOTRH (Kenya), and Brandenburg University of Technology Cottbus–Senftenberg (Germany), and a research permit was granted by the National Commission for Science, Technology, and Innovation, Kenya. To ensure confidentiality, each participant was assigned a unique identification code used in place of names or other identifiers in all data handling, analysis, and reporting. The participants were fully informed of the study’s objectives, data handling procedures, and privacy safeguards prior to participation. Informed consent was obtained in writing only after the participants confirmed their understanding, and the participants retained the right to withdraw at any stage without consequence. Ethical standards were upheld throughout, consistent with national regulations and international guidelines.

## Results

### Health system and institutional barriers

Stakeholders highlighted systemic gaps and resource constraints. A private clinician noted insured patients still face “major challenges [such as] cost…glucometers and test strips are expensive…power supply is unreliable…stock-outs…[and] limited insurance coverage” (K1). Public clinicians described infrastructure shortfalls, stating many GDM patients “don’t attend clinics regularly” due to transport or financial barriers, while some drugs “are not available in the hospital and must be purchased elsewhere” (J4). One private doctor advocated for “universal health coverage…ensuring access to medications, diagnostics, and education” (K1), while another proposed redirecting insurance funds to prevent hospital underresourcing (K2). Even middle-class insured patients faced cost barriers; a clinician explained that “even for insured patients,” monitoring supplies and insulin remain costly (K1), and another physician called for NHIF reforms to address inequitable funding (e.g., hospitals receiving only 20% of collected fees) (K2). Private providers also recommended periodic subcounty GDM clinics to “bring care closer to patients” in rural areas (K1, K2). Public respondents underscored patient poverty; a nurse noted insulin is free only with NHIF coverage: “If she had insurance…it covered the cost. Otherwise, she had to pay” (J2), while many patients “lack the financial means” to sustain treatment (J3). Distributing glucometers to low-income patients was urged (J3). A public patient also cited infrastructure issues, including a maternity ward at JOOTRH that “lacked water,” complicating inpatient care (JP). One affluent patient insisted on a glucose test after her doctor initially “didn’t think I needed” it, ultimately paying for a glucose tolerance test (GTT) privately. She urged facilities to “offer GTT tests and encourage women to take them,” noting supplies like test strips are “expensive…not affordable…especially in rural areas” (KP). The public-sector patient described confusion over unexpected antibiotics and inadequate explanations, stressing that “testing should be standard at clinics” and patients “need clear explanations about what they are being treated for” (JP). These accounts reveal gaps in patient education and counselling. Both sectors stressed improving coverage (e.g., public clinics screening only symptomatic patients) and endorsed system-level solutions like universal health coverage (UHC), expanded insurance, and better funding/education (K1, K2, J4, JP). Some HCWs noted GDM’s infrequency in the MHCS, which hindered consistent management strategies and practical expertise (J1, J3, K2, K3). Providers with greater experience reported more positive attitudes toward GDM care than those with under one year of experience. The small number of GDM patients during the study may itself reflect these systemic and screening-related constraints rather than a true absence of cases; this apparent rarity is considered further in the Discussion.

### Economic and accessibility barriers

Costs and distance were critical barriers. Providers and patients cited high out-of-pocket expenses, with glucometer kits and clinic visits often unaffordable (K1, KP). A public nurse noted many pregnant women “lack the financial means” for treatment and transport (J3), and frequently skipping appointments for financial or transport reasons (J4). Insurance gaps persisted: one nurse emphasized insulin is free only with NHIF coverage (J2), leaving uninsured patients to self-pay. A private doctor highlighted that “monitoring supplies…are expensive,” with some insurers excluding chronic conditions (K1). Another stressed the need for expanded social insurance, citing Kenya’s many working-class or unemployed families who “can’t afford healthcare” without stronger NHIF support (K2). Long travel distances further burdened patients, with mothers often travelling from multiple counties at significant cost (K1).

Public respondents described resource scarcity. A midwife stated women avoid clinics due to transport costs and fees (J4), while drug stock-outs force patients to purchase medications externally (J3, J4). Staff proposed government-distributed glucometers and expanded rural services (J3, J4). The private patient paid privately for a GTT test (unavailable at her clinic) and noted supplies like glucose strips “are not affordable for everyone, especially in rural areas” (KP). Though she could self-monitor, others skip care due to cost. A public-sector respondent described indirect burdens, such as a week-long hospital stay without water, stressing that testing “should be routine” and universally free (JP). Both patients underscored systemic inequities: while some private patients afford supplies, rural or uninsured women are often left behind. Providers universally endorsed affordable care and expanded insurance for low-income families.

### Clinical practices and service delivery

Providers described how GDM care was organized. Private facilities followed standard antenatal care (ANC) protocols: a specialist reported screening “all mothers…for blood sugar during antenatal visits” (K1) via HbA1c and the oral glucose tolerance test (OGTT). Newly diagnosed patients received counselling, insulin if needed, and obstetrician-led follow-up until delivery. Care was “patient-centered,” with dietary guidance and self-monitoring training (K1). Public facilities used ANC profiles to flag GDM but reported varied protocols, including urinalysis and random blood sugar tests. In private settings, GDM patients are hospitalized, stabilized with multidisciplinary care, and discharged with medications and follow-up plans (K1, K4). Nurses reinforced self-care through layered education: “the doctor first explains, then the nurse reinforces…medication use, lifestyle, and diet” (K4). However, one nurse noted guidelines “exist in books” but lack “step-by-step” implementation (K4). Public treatments aligned with global standards, metformin/glibenclamide for mild cases and insulin for severe hyperglycemia (J1, J3), with emphasis on nutrition and exercise. A nurse advised avoiding “sugary foods, fatty foods, alcohol…overly salty foods” (J2), and clinicians educated patients on glucose testing and danger signs (J2, J4). Patient experiences diverged: a private patient with prior GDM self-managed via diet and four daily glucose checks, advocating for specialist input (e.g., a nutritionist’s “very helpful” guidance) (KP). Conversely, a public-sector patient received inpatient glucose testing and unexpected antibiotics (“they told me [these] would help with diabetes”) but no post-discharge monitoring (JP). Both hospitals prioritized early detection, patient education, and systematic screening, with insulin and diet as primary interventions. Follow-up was critical: private nurses promoted coordinated continuous care, while public staff promoted regular ANC health talks to raise awareness.

### Health literacy, communication, and beliefs

Awareness of GDM was uneven. One doctor observed that while general awareness has grown, much of it is from unreliable: “Dr. Google” sites (K1). Language differences were a minor issue in private the setting (the same doctor said miscommunication is rare, except when technical terms were not simplified (K1)). In contrast, public providers felt that language could be a barrier: one nurse agreed that tribal dialects sometimes require an interpreter (J2) and that the labor ward reports relying on relatives or sign-language services for non-Luo patients (J4). Private-setting communication used English/Swahili with local dialects as needed, and one HCW reported occasional miscommunication when jargon was used (K1). In regard to cultural beliefs, one HCW mentioned that patients trust prayer or home remedies over treatment, “some rely on prayers or seek second opinions, doubting the diagnosis” (K1). Misconceptions from the internet were also mentioned: “much of the information is inaccurate”, so counselling is needed (K1). Public providers emphasized the need for patient education. Two nurses noted that not all HCWs routinely screen for or discuss diabetes, so GDM often goes unnoticed (J2, J4). Language was managed by ad hoc means: a public nurse said that for deaf or nonverbal patients, they use interpreters, and for other languages, they find a staff member or family to translate (J2). The insured private patient was proactive and well informed, partly because her family had a history of T2DM. She researched extensively and felt “empowered” to control her condition once she learned that it could be managed with lifestyle changes. She trusted medical sources (valuing her doctors and nutritionist) but also brought her own knowledge, setting stricter glucose targets for herself than her doctor did (KP). In contrast, public-sector patients had low prior awareness. She admitted, “I didn’t think I could have [GDM] because I lacked awareness”, and now believes testing should be standard. She felt undercounseled: “In public facilities, you don’t always get detailed explanations”, so she sought information outside the hospital. Her experience highlights a communication gap: she wanted clear information (even suggesting peer educators at ANC) (JP). All the stakeholders agreed that education is key. Both private and public nurses stressed that caregivers must teach patients about diet, symptoms, and monitoring (e.g., one public nurse listed specific dietary advice, and private nurses reinforced medication use). This underscores the providers’ calls for more community sensitization and patient-directed resources.

### Psychosocial and emotional factors

The diagnosis of GDM elicited strong emotions. Both private and public clinicians repeatedly mentioned shock and anxiety. A private HCW said mothers “initially…go through stages of grief: denial, bargaining, acceptance” when told they have GDM (K1). Nurses in both sectors noted that patients are often “surprised” or “shocked” and sometimes cry upon diagnosis (K4, J1, J4). Patients also expressed fear for their babies: the public patient was “especially afraid I might deliver before term” (JP), mirroring providers’ perceptions (J1). A private provider mentioned needle anxiety and denial noting that starting insulin is difficult because “patients fear needles…self-injection can be overwhelming” (K1). A nurse noted that early denial is common, but patients usually adjust with education (J4). Some providers mentioned nonadherence: a public nurse recalled a patient who “did not follow instructions” and even left her medication at home, likely due to fear or resistance (J1). Public providers mentioned counselling reluctant patients, with one nurse eventually getting her patient to adhere after explaining complications (J2). In challenging cases (one patient lost her baby), staff members found that “she did not open up” and, even when advised, “did not follow instructions” (J1). These examples suggest psychosocial barriers such as denial or mistrust. To combat this, nurses provide reassurance, reminding women that GDM is manageable and not “hopeless” (J1). The private patient’s journey moved from initial regret to empowerment. At first, she was “more shocked” and thought, “Why did I let it get this far?” She worried about complications but focused on learning how to manage them. Once on a diet and exercise plan, she “felt empowered” by weight loss and control. Her husband’s support, joining dietary changes and even refraining from gift chocolates, helped her cope (KP). The public-sector mother also felt distress. She became “confused and stressed” on diagnosis but felt somewhat comforted when providers reassured her that it could be controlled. She did not report stigma or negative reactions from others (no one blamed her, and friends encouraged her diet). After delivery, she experienced less fatigue and nausea because of her lifestyle changes (JP). Across all groups, an initial emotional shock was common. Both doctors and nurses try to counsel and reassure women (for example, repeatedly emphasizing healthy outcomes). Social support emerged as important: the private patient relied on her husband, whereas the public patient leaned on friends. Providers sometimes enlisted family members (one nurse involved a diabetic mother to counsel her daughter (J2)) to bolster understanding and adherence. The survey responses also highlighted positive social support as a key facilitator.

### Sociocultural and household context

Family and community factors shaped each woman’s journey. In the private setting, GDM management became a household effort: the private patient changed family meals (more vegetables, fewer carbs) to support her diet. She noted challenges when attending social events (where foods such as “chapatis, rice, ugali” were served). However, her husband was very supportive (he attended appointments and adapted his gifts to avoid sweets). She reported no negative comments from others about her condition (KP).

In contrast, the public patient was single and without a partner. She lived in a crowded Manyatta slum with safety concerns but found support from a friend who accompanied her to the hospital. She felt responsible for supporting her three children but did not mention any family involvement in her care (JP). The nurses at JOOTRH had to actively gather “next-of-kin” contacts to aid communication. They consider the family context in adherence: one suggested understanding a mother’s support system (husband, relatives) to guide counselling and make referrals to local clinics (J4). Both sectors recognized the influence of family on treatment success (involving relatives in education and follow-up).

### Patient–provider relationship and communication quality

Interactions between patients and clinicians influenced trust and satisfaction. Clinicians emphasized empathy: a public nurse advised colleagues to “understand that [patients] may be in pain”, avoid rudeness, and explain delays or procedures to keep patients calm (J4). A public nurse reassured women with GDM they “can still raise children and achieve their goals” (J1). Private providers similarly stressed education and support, reinforcing instructions over multiple encounters (K1, K4). They spoke little about cultural rifts; one nurse said most patients prioritize respectful treatment over the provider’s tribe (K4), focusing on clear communication. If medical terms confused women, staff explained them simply (K1). They did not report overt mistrust, but one noted patients might compare doctors to internet advice (K1). Public providers noted some patients initially resisted advice, requiring extra counselling; after one pregnant woman refused insulin, a nurse persisted with support until “she eventually accepted it” (J2). In challenging cases, nurses described patients as “difficult” and noncooperative (J1, J4), highlighting the importance of rapport. Some providers relied on family translators to bridge gaps, implicitly trusting that communication aides improved relationships (J2, J4).

The private patient expressed ambivalence about her doctor. She acknowledged his experience and competence but felt he underestimated her high-risk status; he “was relaxed to help me feel relaxed, but that had the opposite effect”. She felt uneasy his glucose targets exceeded what she believed safe, suggesting a subtle trust issue: she continued treatment but self-monitored aggressively due to anxiety (KP). The public patient criticized communication and attitudes. She complained staff gave no detailed test explanations: “Most information I got was later from a gynecologist outside the hospital”. Nurses’ bedside manner also annoyed her, especially being awakened for tests without empathy. She stated that “attitude plays a key role in patient care” and needed improvement, echoing the public midwife’s emphasis on respectful, timely care to build trust (KP, J4). Good provider support was crucial for all. Public and private respondents described taking time to educate and reassure patients (e.g., a JOOTRH nurse explained risk factors and insulin administration (J1)). Understanding improved patient response. Patients confirmed this: the private patient thrived when her husband attended visits and a nutritionist was provided, while the public patient insisted more staff education and kindness would improve her care experience (KP, JP).

### Self-management and lifestyle adaptation

Both clinicians and patients emphasized diet, exercise, and monitoring as central to managing GDM. Clinicians taught patients to self-monitor blood sugar and adjust lifestyles; for example, a private doctor said they “educate patients to monitor their sugar levels, use glucometers…manage their diet, and self-administer insulin if needed” (K1). In private settings, HCWs provide detailed dietary counselling and insulin training (K4). Public providers likewise instructed patients on diet and signs of hypo-/hyperglycemia (J2, J3), often stressing affordable changes (walking, fruit intake) over costly interventions. One private nurse taught women to test blood glucose at home or a local clinic. When prescribing insulin, she personally demonstrated the first injections and then supervised self-injecting. These efforts seemed effective: she reported “most patients had good outcomes, and the diabetes often resolved after delivery” (K3). A public nurse instructed her patient to check sugars “frequently”, recognize high/low symptoms, follow medications, and exercise carefully. She noted one woman struggled with diet but improved with counselling (J2). A public nurse suggested distributing glucometers to poor patients for monitoring (J3). The private patient implemented rigorous lifestyle changes. She tested blood sugar four times daily with a personal glucometer, shifted to a very low-carb, high-protein diet (using apple cider vinegar and almonds to control glucose), exercised as able (though back pain limited her), walked daily when possible, and lost significant weight. These habits managed her GDM and were intended to continue postpartum (KP). The public patient followed guided lifestyle adjustments like walking daily and eating whole fruits (bananas, grapes, pears). With no glucose monitoring post-discharge, she continued exercising (3 km per day) and consuming fruit independently. She noted these changes relieved pregnancy fatigue and nausea (JP). All agreed empowering women to manage their condition at home is vital. Diet education was universal: providers repeatedly teach nutritional balance (e.g., plenty of vegetables, avoiding sugar) (J3, K4). Patients responded positively: the private patient lost weight and felt better (KP), and the public patient walked daily and ate better, with good effect (JP). Both groups saw self-management as essential.

### Public health engagement and system improvement

Most interviewees proposed broader interventions beyond individual care, commonly suggesting increased screening, education, and support programs. Private clinicians repeatedly called for policy changes: one doctor argued for universal screening, health coverage, and more community health workers educating pregnant women (K1, K2). A private physician advocated for stronger county and national support (e.g., directing funds back into hospitals and subsidizing rural clinics) (K2). Private providers favored large-scale health system strengthening: universal health insurance, more staff (doctors, nutritionists, educators), and continuum-of-care models linking community and facility-based services (K1, K2, K3). Public providers stressed outreach and education. A public nurse recommended regular community check-ups and diabetes education, especially in remote areas, “organizing community outreach to educate people on diabetes and conduct checkups” (J3). They proposed recruiting more community health workers for screening and distributing glucometers to low-income mothers (J2, J3). All facilities already give ANC health talks, but public staff want more GDM focus: one nurse noted that continuing antenatal counselling (e.g., on diet and red flags) is crucial for awareness (J4). The private-sector patient urged that maternity hospitals provide onsite GDM testing (she travelled out of the hospital to Lancet for her OGTT) and integrate nutritionists into routine care (her first doctor’s referral to a dietitian was “very helpful”). She also highlighted accessibility: affordable glucometer supplies should be available, and GDM education should be scaled up (KP). The public patient advocated for better education, communication, and improved nurses’ attitudes. Importantly, she argued that “testing should be routine” at clinics, reflecting a public health push for mandatory GDM screening. She also recommended peer-led education at ANC, indicating that hearing from fellow mothers could raise awareness faster than hearing from clinical staff alone (JP). Everyone saw GDM as a public health issue requiring systemic action. Providers and patients from both sectors favored routine ANC screening (many said it’s “already part of the profile” or should be). Education was another common theme: all groups called for more patient-facing information, whether via brochures, seminars, community health workers, or peer educators. In summary, findings suggest that while private and public settings differ in resources and clientele, there is universal agreement on strengthening the health system (through insurance, staffing, and community outreach) to improve GDM care. Table 5 presents selected illustrative examples and direct quotations from women and HCWs in both public and private healthcare settings in Kisumu, Kenya. The quotations are organized by key themes reflecting barriers to and facilitators of GDM service utilization, as identified in qualitative interviews. Data highlight systemic, financial, cultural, and knowledge-related barriers to GDM care, alongside facilitators such as patient support systems and integrated care models.

Questionnaire responses from women with a history of GDM at the public hospital reinforced these themes. All respondents underwent GDM screening during pregnancy, many receiving medical management with insulin or metformin. None reported using traditional or alternative medicine, indicating dominant reliance on biomedical care. However, knowledge gaps were evident regarding risk factors, symptoms, and long-term implications. While most identified common risk factors like family history of diabetes, many were confused about conditions such as polycystic ovary syndrome and physiological mechanisms. Similarly, while they recognized classic symptoms like excessive thirst and frequent urination, some incorrectly associated unrelated symptoms (e.g., mood swings) with GDM. Despite these gaps, all respondents acknowledged the importance of lifestyle modifications (diet, exercise). Social support emerged as critical, with every woman affirming that help from family or friends aided coping. Most participants understood GDM increased their future T2DM risk, highlighting the need for continued education and follow-up care postpartum. These findings should be interpreted with caution: the surveyed women were already engaged with the healthcare system and relatively well educated, potentially yielding more informed responses than women with GDM not accessing tertiary care.

## Discussion

Routine screening is critical for early GDM detection. HCWs emphasized systematic ANC screening to detect GDM alongside conditions like hypertension and preeclampsia, consistent with research showing integrated screening increases early detection and enables timely intervention [22, 27, 29, 41]. However, one study found that early screening of high-risk women did not reduce cesarean deliveries, preeclampsia, macrosomia, or birth injuries. Compared with standard screening, those screened before 20 weeks had higher insulin requirements and preterm birth rates, likely reflecting greater prevalence of elevated body mass index, prior GDM, and chronic hypertension, suggesting early screening identifies women with severe metabolic risks needing intensive management [42]. HCWs stressed integrating GDM screening into broader ANC practices aligned with World Health Organization recommendations for comprehensive ANC packages to optimize outcomes [43]. Universal screening is a cornerstone of UHC, which was prioritized to address financial barriers, as evidence from other LMICs shows it improves access to essential services and reduces out-of-pocket expenses. However, achieving UHC requires equitable access, sufficient resources, financial sustainability, and strong political commitment [44–47].

Reported GDM prevalence and incidence depend on the diagnostic criteria and testing modality applied, complicating comparison across studies and likely reflected in the heterogeneous practices observed here. Several thresholds coexist internationally, including the one-step 75 g OGTT recommended by the International Association of Diabetes and Pregnancy Study Groups (IADPSG) and adopted by the World Health Organization in 2013, the earlier World Health Organization 1999 criteria, the two-step approach using a 50 g glucose challenge followed by a 100 g OGTT with Carpenter–Coustan or National Diabetes Data Group cut-offs, and the more restrictive National Institute for Health and Care Excellence thresholds. Because these definitions apply different glucose loads and cut-offs, the same population can yield substantially different prevalence estimates, with the one-step IADPSG approach generally classifying more women as having GDM than two-step strategies [2, 5]. In the present study, the private facility screened antenatal attendees using HbA1c and the OGTT, whereas public facilities relied on more variable approaches that included urinalysis and random blood sugar. These differences matter: random blood sugar and urinalysis are insensitive relative to OGTT or HbA1c and under-detect milder hyperglycaemia, so the few recognised cases in this and comparable Kenyan settings may partly reflect non-standardised, less sensitive testing rather than a genuinely low burden. The absence of a single, consistently applied national protocol therefore contributes both to the likely underestimation of GDM and to the inconsistent advice that patients reported receiving across facilities.

HCWs noted that collaboration among obstetricians, gynecologists, physicians, and laboratory staff facilitated comprehensive care. This interdisciplinary approach aligns with literature showing collaboration improves outcomes: reviews link teamwork to better evidence-based practice exchange, decision-making, innovation, shorter hospital stays, and stronger guideline adherence. Collaborative practice also increases responsiveness, efficiency, and patient-centered care, and supports engagement through education, shared decision-making, and self-care [48, 49]. Nevertheless, this study did not directly assess care quality, the nature of interprofessional collaboration, or HCW availability/training. Some studies show these factors influence collaboration quality [50–53]. Existing data indicate a persistent shortage of trained HCWs in Kenya, with pronounced urban-rural disparities [7, 20, 21, 54].

Patient education and self-management support were crucial for GDM outcomes. HCWs emphasized teaching patients self-monitoring of blood glucose, dietary modification, insulin administration, and lifestyle change. Studies show patient education improves adherence and reduces complications [55, 56]. Despite understanding common risks like obesity and family history, gaps remain regarding less common risks such as polycystic ovary syndrome and hypertension. Barriers include low awareness, negative self-management attitudes, socioeconomic challenges, limited resource access, and shortages of skilled HCWs and community support. Consistent with our results, poor communication and negative provider attitudes, particularly where patients attended visits alone, undermined trust and adherence. Addressing these requires better communication and supportive care environments to improve GDM service accessibility and effectiveness [7, 57].

Social support from spouses, family, and communities was instrumental in coping with GDM’s emotional burden and adhering to treatment, consistent with literature on social networks in chronic disease management, where emotional and practical support significantly improves outcomes [7, 58]. The study’s finding that language barriers were mitigated through hospital staff, family, and sign language experts aligns with literature recognising language concordance as a foundational determinant of equitable care, though it invites nuance about who interprets [59–61]. A systematic review of 21 studies found that professionally trained medical interpreters were associated with fewer clinically significant communication errors, better patient comprehension, more equitable service utilisation, improved clinical outcomes, and higher satisfaction than ad hoc interpreters such as family members or untrained bilingual staff [59]. Another study of multiethnic obstetric care in London reached convergent conclusions: immigrant women and providers identified shared language, rather than ethnic or gender concordance, as central to a sensitive clinical encounter, yet interpreter services operated suboptimally owing to issues of quality, trust, accessibility, and absent standardised training [60]. The use of family members, while pragmatic, was further associated with breaches of confidentiality and emotional harm [60]. Against this evidence, the reliance on a mix of hospital staff, family, and sign language experts is a partial rather than complete solution. For deaf and nonverbal patients, involving qualified sign language professionals is consistent with best practice; for spoken-language needs, reliance on family or untrained staff is a recognised vulnerability. Because this study did not distinguish trained professional interpreters from ad hoc translators, the proportion of encounters supported by qualified interpreters cannot be determined. The finding that health insurance was a critical determinant of service accessibility is consistent with the wider literature on healthcare access in LMICs. In a nationally representative analysis of 13 266 Tanzanian women, a study demonstrated additive effects of socioeconomic barriers, with insured women having 0.62 times the odds of falling into higher problem-accumulation categories, suggesting insurance buffers against the clustering of barriers that typically deter utilisation [61]. Evidence from Kenya showed only 7% of reproductive-age women held any health insurance, with coverage strongly patterned by formal-sector employment, wealth, education, and urban residence, systematically excluding the poor, the informally employed, and rural women [62]. Another study found that providers across 26 LMICs reported out-of-pocket costs for diagnostic tests, ultrasound, glucometers, test strips, insulin, and recommended diets routinely prevented women from completing screening and follow-up, prompting the question, “Why screen if we cannot follow-up and manage? [49]. A study documented that, even after Kenya’s 2013 abolition of user fees for maternity services, indirect costs (transport, ambulance fuel, and consumables) continued to perpetuate inequities in facility-based care [22]. The finding that infrequent GDM encounters undermine provider competence aligns with comparable settings. In a survey of 67 providers across 26 LMICs, clinicians frequently lacked GDM-specific knowledge, received no structured training, and screened inconsistently, some screening only women with overt risk factors and others omitting screening even when such factors were present [49]. This pattern mirrors the clinical hesitancy and weakened guideline adherence found here. These data also support the concern that reported incidence understates true prevalence: distance, cost, late antenatal booking, and the concentration of diagnostic services in tertiary or private facilities systematically prevent affected women from reaching care, producing an artificially low caseload that further limits provider exposure [49]. Furthermore, the low observed incidence likely reflects systemic screening and reporting gaps and COVID-19 disruptions to maternal health service utilization [63].

Policy experts in the first phase identified gaps in GDM data collection, aggregation, and reporting as central barriers in Kenya. Limited resources constrain standardized register distribution, leaving most data in facility-level paper records and causing inconsistent, incomplete reporting that fails to inform national understanding. Bureaucratic barriers and the lack of routine quality assurance further weaken data quality, hindering gap identification and resource allocation. For HCWs, infrequent GDM diagnosis (driven by underreporting and insufficient data) creates a false perception of rarity, reducing screening vigilance and limiting skill development, perpetuating underdiagnosis and undermining provider engagement and outcomes. Although systemic barriers persist, current MoH efforts to digitize and integrate health information systems, alongside targeted data quality investments, offer opportunities to strengthen GDM service delivery and improve maternal and child health. Geographical and transport-related barriers significantly impeded GDM service access, particularly for rural women, with multiple studies identifying transportation, location, and distance as key barriers to MHCS access in LMICs [22, 27, 49, 64–66]. The barriers identified in Kisumu echo those documented elsewhere in Kenya and other low-resource settings, suggesting they are not unique to the study county. A qualitative study in two contrasting Kenyan counties (Kilifi and Kisii) similarly found staff shortages, supply stock-outs, long travel distances, poor transport infrastructure, financial constraints, limited family support, and disrespectful provider attitudes as dominant obstacles to facility-based maternity care, despite the removal of user fees [22]. Nationally, persistent understaffing, underfunding, and drug stock-outs following the 2013 devolution of health services have been described as structural features of the Kenyan system rather than a local anomaly [20]. Although our sample was drawn from a single county, the core barriers and facilitators identified are likely to be broadly transferable; what is more likely to vary between contexts is their relative weight and the specific policy levers available (for example, county-level financing arrangements and the ongoing transition from the NHIF to the Social Health Authority).

### Methodological strengths and limitations

#### Strengths

The study used a multistakeholder, multimethod approach, interviewing HCWs and patients across public and private sectors and supplementing interviews with structured survey data. This triangulation enabled credible, in-depth interpretation by allowing verification across diverse perspectives and data sources.

Data collection instruments were systematically developed using established frameworks (HNA and KAB), strengthening construct validity and ensuring comprehensive coverage of systemic and individual-level GDM care determinants. To minimize bias and enhance credibility, they were reviewed and validated by a multidisciplinary Kenyan-German expert panel for content validity and cultural appropriateness.

Qualitative data were independently double-coded by two researchers, with discrepancies resolved through structured consensus discussion. Member checking confirmed thematic accuracy through participant feedback, while peer debriefing with independent experts strengthened analytical rigor and reflexivity. The team maintained reflexive practices throughout, considering the potential influence of positionality on data collection and interpretation.

This study builds on prior qualitative research with Kenyan MoH policy experts that identified GDM epidemiological data challenges and key care barriers and facilitators. These insights were triangulated with HCW and patient perspectives for a comprehensive contextual understanding. The challenges and themes identified are likely analogous to other low-resource settings, underscoring broader relevance.

#### Limitations

This study has several limitations to consider when interpreting the findings. The sample was modest, comprising interviews with eight healthcare workers and two women with GDM and surveys from eleven women with a history of GDM, all of whom were recruited from Kisumu County and predominantly engaged with tertiary or referral-level care. Data collection was confined to two facilities within a single county, which may not capture the full spectrum of health system variation, particularly in rural or remote areas where barriers to GDM care may differ substantially. Consequently, generalizability is limited, especially for women outside major counties and facilities or not accessing formal care. Data saturation is not claimed for the patient strand, and the two patient interviews are best read as illustrative cases rather than a thematic sample. By the same token, contrasts drawn between the “private” and “public” patient throughout the results are based on a single participant in each setting; these individuals differed not only by facility type but also by socioeconomic status, education, insurance, parity and household structure, any of which could account for the observed differences. The HCW sample, although evenly drawn from the two facilities, comprised a mix of cadres and experience levels that was too small to support inferences about how role or seniority shapes provider perspectives. Nonetheless, the convergence between our findings and those from other Kenyan counties and multi-country surveys of low- and middle-income settings (see Discussion) suggests the principal barriers and facilitators are unlikely to be confined to Kisumu, even if their relative importance and the most appropriate local solutions differ between counties. The facilities also applied heterogeneous and, in the public sector, non-standardised screening approaches, which constrains inference about true local GDM prevalence or incidence and reinforces the need for cautious interpretation of the apparent rarity of cases.

Interviews and surveys relied on self-reported data, risking recall and social desirability bias regarding health-seeking behaviors, knowledge, and adherence. Member checking was limited to three HCWs and did not include patients, so participant validation of patient-derived themes was not obtained. Purposive sampling, pragmatic given bureaucratic and logistical challenges, may have overrepresented informed, engaged, or accessible participants while excluding women not seeking care or diagnosed outside formal facilities, potentially underestimating barriers for marginalized groups. The COVID-19 pandemic further constrained recruitment, as measures such as lockdowns and curfews reduced facility attendance and disrupted routine ANC, also contributing to a low number of GDM cases present during the study period and required the research team to adapt by recruiting only those able and willing to access care at the selected facilities. As a result, women who faced additional barriers, such as fears related to COVID-19, transportation difficulties, or economic hardship, may have been unintentionally excluded.

Future research could address these limitations by enlarging and diversifying samples through multi-county recruitment (including rural/lower-level facilities), partnering with community health workers to reach women not accessing formal healthcare, employing longitudinal mixed-methods designs to assess long-term outcomes and GDM sequelae, and leveraging digital tools for remote data collection to improve inclusivity during health emergencies or restricted facility access.

## Conclusions

In summary, this study suggests that financial barriers, resource shortages, and inconsistent clinical protocols constrain access to GDM care in the settings studied, whereas strong social support and effective provider communication appeared to facilitate better management. These findings underscore the need for health system reforms, including expanded insurance coverage, improved supply chains for essential diagnostics and medications, standardized GDM protocols, and enhanced provider training to promote patient-centered care. Despite the study’s strengths, such as its multimethod design and rigorous analysis, its limitations include the use of a small, facility-based sample and potential recruitment biases exacerbated by the COVID-19 pandemic, which may restrict generalizability. Nevertheless, the results contribute to the evidence base that aims to inform policymakers and practitioners that routine GDM screening, targeted education campaigns, and greater community and family engagement are essential for improving maternal health outcomes. Future research should broaden participant diversity, include rural and lower-level facilities, and assess the long-term impact of interventions. Ultimately, strengthening GDM services will advance health equity and maternal‒child health, both in Kenya and comparable low-resource settings.

**Table 5:** Illustrative quotations on barriers to and facilitators of GDM service utilization in Kenya, from healthcare worker and patient perspectives

Theme	Sub-theme	Participant	Illustrative quotation	Context
Barriers				
Financial constraints	Cost of diagnostics	Private Patient (KP)	*“Supplies like glucose strips are expensive. A glucometer and strips aren’t affordable for everyone*,* especially in rural areas.”*	High out-of-pocket costs for self-monitoring tools.
	Medication access	Public HCW 4 (J4)	*“Some drugs are not available in the hospital and must be purchased elsewhere*,* which many cannot afford… Some are single mothers or widows struggling economically.”*	Stockouts and poverty limiting treatment adherence.
Health system gaps	Inconsistent screening	Public HCW 4 (J4)	*“Full screening for diabetes is only done on doctor’s request*,* based on symptoms. No universal GDM screening is done.”*	Selective screening misses at-risk women.
	Policy/funding flaws	Private HCW 2 (K2)	*“Funds collected through healthcare… are remitted to counties*,* which may allocate only a fraction back to hospitals… A hospital collects KES 100*,*000*,* but the county gives back KES 20*,*000.”*	Systemic underfunding undermining service delivery.
Knowledge & awareness	Patient misconceptions	Private HCW 1 (K1)	*“Awareness has grown thanks to social media… But much information is inaccurate—‘Dr. Google’ is a common culprit. Many misconceptions exist.”*	Misinformation complicating patient education.
	Provider preparedness	Private HCW 3 (K3)	*“We haven’t had a GDM case here… but staff are informed and ready.”* *“I haven’t personally handled insulin use during pregnancy.”*	Limited hands-on experience affecting confidence in care.
Cultural & communication	Language barriers	Private HCW 4 (K4)	*“Language barriers exist when patients only understand vernacular. We need translators*,* especially in community centers.”*	Communication challenges in multilingual settings.
	Dietary adherence	Public HCW 2 (J2)	*“It was difficult for her to adjust to dietary changes—like switching from white to brown bread and white to brown ugali.”*	Cultural dietary preferences hindering management.
Infrastructure	Resource limitations	Private HCW 1 (K1)	*“Insulin needs refrigeration*,* but power supply is unreliable… Compatibility issues between glucometers and test strips*,* stock-outs*,* travel distances.”*	Weak infrastructure complicating diabetes management.
Facilitators				
Patient support systems	Family involvement	Public HCW 2 (J2)	*“After taking her history*,* I learned her mother was diabetic*,* so I involved the mother in counseling her.”*	Leveraging family support to improve adherence.
	Partner support	Private Patient (KP)	*“My husband was supportive… For Valentine’s Day*,* I asked him not to get chocolate. He supported me in small but meaningful ways.”*	Emotional/practical support enhancing self-care.
Integrated care models	Multidisciplinary teams	Private HCW 4 (K4)	*“Education is provided at two levels: the doctor explains*,* then the nurse reinforces… Pharmacy and lab staff add support during interactions.”*	Team-based care improving patient understanding.
Education & empowerment	Self-management training	Private HCW 3 (K3)	*“We teach those with monitoring kits how to test blood sugar and interpret results… With follow-up*,* they eventually manage well.”*	Training enabling patient autonomy.
Proposed solutions				
System strengthening	Universal screening	Private HCW 2 (K2)	*“Universal screening is appropriate… We’re changing*,* our diets*,* our lifestyles.”* *“Glucose testing is part of ANC profiles*,* so we’re already doing it.”*	Advocacy for routine screening integrated into ANC.
	Community outreach	Public HCW 3 (J3)	*“Organize community outreach to educate people on diabetes… Deploy more staff to remote areas for screening and education.”*	Bridging urban-rural gaps in access.
Policy advocacy	Financing reform	Private HCW 2 (K2)	*“Universal healthcare is coming*,* but we need a strong social scheme like NHIF to cater for those with no steady income… Pooled resources can support the ill.”*	Call for equitable financing mechanisms.
Patient-centered care	Empathetic communication	Public HCW 4 (J4)	*“Understand that patients may be in pain*,* not rude. Explain delays and give reassurance… Treat them with respect to make them feel seen.”*	Humanizing care to build trust and cooperation.

Table 5 presents key themes, sub-themes, and illustrative quotations extracted from interviews with HCWs and patients across public (JOOTRH) and private (KSH) Kenyan health facilities

## Data Availability

The datasets generated and/or analysed during the current study are not publicly available to protect participant anonymity and confidentiality but are available from the corresponding author upon reasonable request.

## Abbreviations

ANC: Antenatal Care
AO: Angela Obongo (Principal investigator)
GDM: Gestational Diabetes Mellitus
GTT: Glucose Tolerance Test
HE: Hannah Eger (Co-author)
HCW(s): Healthcare Worker(s)
HNA: Health Needs Assessment
IADPSG: International Association of Diabetes and Pregnancy Study Groups
IDF: International Diabetes Federation
JOOTRH: Jaramogi Oginga Odinga Teaching and Referral Hospital
J1–J4: Jaramogi Oginga Odinga Teaching and Referral Hospital healthcare workers (*n* = 4)
JP: Jaramogi Oginga Odinga Teaching and Referral Hospital patient with GDM
KAB: Knowledge, Attitude, and Behaviour
KSH: Kisumu Specialists Hospital
K1–K4: Kisumu Specialists Hospital healthcare workers (*n* = 4)
KP: Kisumu Specialists Hospital patient with GDM
LMIC(s): Low- and Middle-Income Country/Countries
MCHS: Maternal and Child Health Services
MoH: Ministry of Health
NCD(s): Non-Communicable Disease(s)
NHIF: National Health Insurance Fund
OGTT: Oral Glucose Tolerance Test
QCA: Qualitative Content Analysis
SHA: Social Health Authority
SSAC(s): Sub-Saharan African Country/Countries
T2DM: Type 2 Diabetes Mellitus
UHC: Universal Health Coverage
